# High salivary levels of JP2 genotype of Aggregatibacter actinomycetemcomitans is associated with clinical attachment loss in Moroccan adolescents

**DOI:** 10.1002/cre2.156

**Published:** 2019-01-24

**Authors:** Oum Keltoum Ennibi, Rolf Claesson, Sanae Akkaoui, Sarah Reddahi, Francis Kwamin, Dorte Haubek, Anders Johansson

**Affiliations:** ^1^ Department of Periodontology, School of Dentistry Mohammed V University Morocco; ^2^ Division of Oral Microbiology Umeå University Sweden; ^3^ Laboratory of Oral Microbiology and Biotechnology School of Dentistry, Mohammed V University in Rabat Morocco; ^4^ Dental School University of Ghana Ghana; ^5^ Section for Pediatric Dentistry, Department of Dentistry and Oral Health Aarhus University Denmark; ^6^ Division of Molecular Periodontology, Department of Odontology Umeå University Sweden

**Keywords:** Aggregatibacter actinomycetemcomitans, interleukin‐1β, JP2 genotype, quantitative PCR, saliva, virulence blocking

## Abstract

It has previously been shown that the presence of *Aggregatibacter actinomycetemcomitans* in subgingival plaque is significantly associated with increased risk for clinical attachment loss. The highly leukotoxic JP2 genotype of this bacterium is frequently detected in adolescents with aggressive forms of periodontitis. The aims of the study were to quantify the levels of JP2 and non‐JP2 genotypes of *A. actinomycetemcomitans* in saliva of Moroccan adolescents with the JP2 genotype earlier detected in the subgingival plaque. The salivary concentrations of inflammatory proteins were quantified and linked to the clinical parameters and microbial findings. Finally, a mouth rinse with leukotoxin‐neutralizing effect was administrated and its effect on the levels the biomarkers and *A. actinomycetemcomitans* examined. The study population consisted of 22 adolescents that previously were found to be positive for the JP2 genotype in subgingival plaque. Periodontal registration and sampling of stimulated saliva was performed at baseline. A mouth rinse (active/placebo) was administrated, and saliva sampling repeated after 2 and 4 weeks rinse. The salivary levels of JP2 and non‐JP2 were analyzed by quantitative PCR and inflammatory proteins by ELISA. Both the JP2 and the non‐JP2 genotype were detected in all individuals with significantly higher levels of the non‐JP2. Enhanced levels of the JP2 genotype of *A. actinomycetemcomitans* was significantly correlated to the presence of attachment loss (≥3 mm). Salivary concentrations of inflammatory biomarkers did not correlate to periodontal condition or levels of *A. actinomycetemcomitans*. The use of active or placebo leukotoxin‐neutralizing mouth rinse did not significantly interfered with the levels of these biomarkers. Saliva is an excellent source for detection of *A. actinomycetemcomitans* on individual basis, and high levels of the JP2 genotype were significantly associated with the presence of clinical attachment loss.

## 1 INTRODUCTION

Periodontal clinical attachment loss (CAL) is highly prevalent in adolescents in North‐ and West‐Africa (Haubek et al., 2001, 2008; Haubek, Ennibi, Poulsen, Benzarti, & Baelum, 2004; Höglund Åberg, Kwamin, Claesson, Johansson, & Haubek, 2012; Kissa et al., 2016). High prevalence of a highly leukotoxic genotype (JP2) of the periopathogen *Aggregatibacter actinomycetemcomitans* has contributed to explain the high numbers of aggressive periodontitis (AgP) in these geographic regions (Haubek et al., 2008; Höglund Åberg et al., 2014). Longitudinal studies have shown that periodontally healthy carriers of the JP2 genotype of *A. actinomycetemcomitans* in the subgingival plaque have a significantly increased risk to develop CAL compared with individuals without this bacterium (Haubek et al., 2008; Höglund Åberg et al., 2014). The leukotoxin that is highly expressed by the JP2 genotype of *A. actinomycetemcomitans* protects the bacteria from phagocytic killing by neutrophils and induces a pro‐inflammatory cell death in macrophages (Johansson, 2011). The leukotoxin‐affected macrophages release substantial amounts of bioactive interleukin‐1β (IL‐1β) by activation of the inflammasome complex in the cytosol (Kelk et al., 2011). This cytokine is a highly active pro‐inflammatory protein that is used as a target in the treatment of many inflammatory‐induced degenerative disorders (Dinarello, Simon, & van der Meer, 2012).

In aggressive forms of periodontitis, CAL occurs at a much faster rate than in chronic periodontitis and the disease is associated with the presence of specific pathogens (Könönen & Müller, 2014). The presence of *A. actinomycetemcomitans* in the subgingival plaque is highly linked to AgP, especially the highly leukotoxic genotypes described previously (Ennibi, Benrachadi, Bouziane, Haubek, & Poulsen, 2012; Haubek et al., 2001, 2008; Höglund Åberg et al., 2014; Johansson, Claesson, Höglund Åberg, Haubek, & Oscarsson, 2017). The management of AgP remains a challenge for the clinicians due to lack of standardized protocols for effective disease control (Akrivopouloua, Green, Donos, Nair, & Ready, 2017). The traditional management of this disease consists on mechanical debridement with or without surgery, and generally supplemented with antimicrobial drugs (Deas & Mealey, 2010; Eick et al., 2018). There is a great need of new more specific treatment strategies as well as new tools for an early identification of risk individuals. Targeting the etiological factors before disease onset might be the optimal preventive strategy for aggressive forms of periodontitis. In this infection‐induced inflammatory disease, both bacterial virulence factors and host‐related inflammatory proteins have the potential to be targets for therapeutic strategies.

Colonization of *A. actinomycetemcomitans* in humans takes place in early childhood, acquired from close relatives and localized in the oral mucosa (Könönen & Müller, 2014). The bacterium can be translocated from the initial oral colonization site to subgingival crevices and has to compete with other bacteria in the periodontal niche. Establishment of persistent colonization of *A. actinomycetemcomitans* in subgingival crevices may lead to periodontal destruction and thereby development of periodontitis in susceptible individuals (Fine, Kaplan, Kachlany, & Schreiner, 2006). This colonization pattern of the bacterium indicates that saliva can be a useful source for microbial sampling in order to identify healthy risk individuals carrying periodontal pathogens.

Plants and other natural products have been extensively used in the management of oral infections and improvement of oral health in many populations (Akkaoui & Ennibi, 2017; Chinsembu, 2016). Among several tested herbal plants, we found that extract of leaves or twigs from Psidium guajava efficiently neutralizes the activity of *A. actinomycetemcomitans* leukotoxin (Kwamin, Gref, Haubek, & Johansson, 2012). Virulence blocking might be a tool for preventive strategies for aggressive forms of periodontitis associated with the presence of highly leukotoxic *A. actinomycetemcomitans* (Ben Lagha, LeBel, & Grenier, 2018; Haubek & Johansson, 2014).

The aims of the present study were to quantitatively examine the presence of JP2 and non‐JP2 genotypes of *A. actinomycetemcomitans* in saliva of healthy as well as periodontally diseased Moroccan adolescents. Second, it was to quantify inflammatory proteins in saliva in relation to the number of *A. actinomycetemcomitans* and the presence of CAL. Finally, we analyzed effect of leukotoxin‐neutralizing mouth rinse on salivary levels of *A. actinomycetemcomitans* and inflammatory proteins.

## 2 MATERIALS AND METHODS

### 2.1 Ethical considerations

Ethical clearance for this study has been approved by the Ethical committee of Mohammed V University, Rabat, Morocco (N/R 67/16). Signed consents were received from the parents or the guardians of the participants before they entered the study. Subjects identified with AgP were informed of their status and referred for treatment to the Dental School of Mohammed V University, Rabat, Morocco.

### 2.2 Study population

The study population comprised 22 Moroccan pupils from different secondary schools in Rabat. All individuals have previously been shown to be positive for the presence of the JP2 genotype of *A. actinomycetemcomitans* in subgingival plaque, when analyzed at the age of 8–9 years by conventional PCR (Jensen, Ennibi, Ismaili, Poulsen, & Haubek, 2016). The examined adolescents consisted of 13 boys and nine girls with a mean age of 13.5 ± 0.7 years. All examined students had a bad oral hygiene. Indeed, they all showed light to heavy plaque deposits on tooth surfaces, and all had mild gingivitis. Spontaneous bleeding was reported by 8.7% of the student, and 78.2% reported having gingival bleeding when they brushed their teeth. In a previous study on oral hygiene in Moroccan school children and their mothers, authors noticed that the use of a toothbrush was low in children (68%); and clinical evaluation of plaque index was high 56.2. In the same study, bleeding index was 13.3 and the average decayed missing filled teeth index was 6.5 ± 2.6 (Assimi, Tajmouti, & Ennibi, 2016).

### 2.3 Collection of saliva samples

Saliva from each participant was collected before the clinical examination, as well as after 2 and 4 weeks of rinsing with the test products. The participants were asked to chew a paraffin tablet (Ivoclar Vivadent AB, Solna, Sweden) for 1 min. Subsequently, saliva (>1 ml) was collected in a plastic beverage cup. One milliliter of the saliva was transferred into a sterile plastic tube with screw cap and stored in a freezer (−20°C) until quantification of *A. actinomycetemcomitans* and inflammatory proteins.

### 2.4 Clinical examination and documentation

The examination included measurement of the probing pocket depth and the distance from the free gingival margin to the cemento‐enamel junction at the buccal aspect of the mesial and distal surfaces of all fully erupted permanent teeth by means of a calibrated periodontal probe (Rönvig®, Kohler, Germany). CAL was defined as the distance from the cemento‐enamel junction to the bottom of the periodontal pocket or crevice and was calculated as the difference between the two measurements described above. Individuals were regarded as being periodontally diseased, if one or more sites had periodontal attachment loss of 3 mm or more, and as periodontally healthy if no such sites were found. After the first examination (Day 0), all received a toothbrush, fluoride toothpaste, and the mouth rinse (either the placebo or the active solution). At the end of the clinical study, the students were given a questionnaire regarding treatment satisfaction. The students were asked questions about the taste of the product and gingival bleeding before and after using the mouth rinse.

### 2.5 Production and administration of active and placebo mouth rinse

Guava leaves were collected in Ghana by Dr F. Kwamin and transported with a courier to Umeå University, Umeå, Sweden. Guava leaves at a concentration of 250 g/L water were boiled for 10 min before the leaves were removed by filtration and cleared from debris by centrifugation. The guava raw extract was mixed in 0.2% sodium fluoride in water at a concentration of 5% of the total volume (active mouth rinse). The placebo mouth rinse consisted of only 0.2% sodium fluoride in water and was packed and labeled identically with the active mouth rinse. Each participant was administrated randomly 2 × 60 ml mouth rinse of either active or placebo mixture. All individuals were instructed to rinse 10 ml twice per day (morning and evening) during a 4‐week period. The manufacturing of the guava mouth rinse clinical material, that is, active test product and placebo test product, has been performed according to a standard close to cGMP (Camber consulting AB, Bromma, Sweden). Leukotoxin‐neutralizing activity and microbial purity of the test products were controlled before the onset of the clinical trial. Both the examiner and the participants were blinded to the allocated mouthwash sample.

### 2.6 qPCR‐based quantification of A. actinomycetemcomitans

#### 2.6.1 DNA isolation

Stimulated saliva was collected from 22 individuals, three samples per donor, and mixed 1:1 with DNA preservation buffer (Norgen Biotek Corporation, Thorold, Canada). The Viral DNA extraction kit (DiaSorin AB, Dublin, Ireland) was used for the DNA isolation, and for the procedure, an automated extraction instrument was used (Liaison® IXT, Diasorin AB, Ireland). DNA was extracted from 550 μl of the sample mixture and eluted in a volume of 100 μl. Standard suspensions of the JP2 genotype (HK1651) and non‐JP2 genotype (D7s), respectively, (10^8^–10^1^ cells/ml), prepared in *A. actinomyctemcomitans*‐free saliva, were treated as described above. The samples and the standard solutions were stored at +4°C until use.

#### 2.6.2 Quantification methods

Quantification of the total concentration of *A. actinomycetemcomitans* in saliva was performed according to Kirakodu, Govindaswami, Novak, Ebersole, and Novak (2008). Briefly, PCR mixture (10 μl) contained 5‐μl Kapa Syber Green (KK 4601; Kapa Biosystems, Boston USA), 4 μl template, and 1 μl of a primer mix specific for the *ltxA* (0.5 μM each; Table 1). The PCR program is shown in Table 2.

**Table 1:** *Aggregatibacter actinomycetemcomitans*‐specific primers according to Kirakodu et al. (2008) and Yoshida et al. (2012)

	Forward	Reverse
Kirakodu		
*ltxA*	CTAGGTATTGCGAAACAATTTG	CCTGAAATTAAGCTGGTAATC
Yoshida		
non‐JP2	CGCAAGTGCCATAGTTATCCACT	TCGTCTGCGTAATAAGCAAGAGAG
JP2	TCTATGAATACTGGAAACTTGTTCAGAAT	GAATAAGATAACCAAACCACAATATCC

**Table 2:** Cycle settings for quantification of *Aggregatibacter actinomycetemcomitans* according to Kirakodu et al. (2008) and Yoshida et al. (2012)

	Kirakodu	Yoshida
Hold/time	95°/10 min	95°/10 min
Cycling/time	95°/10 s	95°/10 s
Cycling/time	55°/5 s	58°/40 s
Cycles	45	45

Quantification of the concentration of JP2 and the non‐JP2 genotypes, respectively, in saliva was performed according to Yoshida et al. (2012). Briefly, PCR mixture (10 μl) contained 5‐μl Kapa Syber Green (KK 4702; Kapa Biosystems, Boston USA), 3‐μl template, and 1 μl of a primer mix targeting JP2 or non‐JP2 genotypes specific sequences within the leukotoxin promoter region of the leukotoxin operon (0.5 μM each; Table 1). The PCR mixtures also contained 1 μl of JP2 or non‐JP2 genotypes related probes (0.2 μM; Table 3). The PCR program is shown in Table 2.

**Table 3:** JP2 and non‐JP specific probes according to Yoshida et al. (2012)

*JP2*	FAM‐ACAAATCGTTGGCATTCTCGGCGAA‐TAMRA
*nJP2*	FAM‐ATATTGTAGACATCGCCC‐MGB

The samples and the standards solution (10^8^–10^1^ cells/ml) were analyzed in duplicates by using a Corbett Research Rotor‐Gene 6000 Rotary Analyze instrument (QIAGEN, Valencia, CA, USA).

The two qPCR‐based methods for quantification of *A. actinomycetemcomitans* in the saliva samples, in accordance with Kirakodu (total Aa) and Yoshida (non JP2 + JP2), respectively, were compared.

### 2.7 Quantification of inflammatory proteins by ELISA

The inflammatory‐associated proteins IL‐1β, MMP‐8, sCD14, and sICAM‐1 were quantified by commercial ELISA kit available from RnD systems (Abingdon, UK). The saliva samples were thawed and an aliquot centrifuged in 10,000*g* for 10 min at 4°C. The supernatants were stored on ice, and the specific proteins in the solutions were quantified by the different ELISA kits in accordance to the manufacturer's protocol (RnD systems).

### 2.8 Statistical analyses

Data analyses were performed using SPSS 24.0 (SPSS Inc., Chicago, IL, USA) and STATA 8.0 (StataCorp LP., College Station, Texas, USA). In the statistical analyses, the primary outcome was salivary levels of *A. actinomycetemcomitans* (JP2 or non‐JP2 genotypes), inflammatory biomarkers, and CAL ≥ 3 mm in one or more sites at the subject level. The quantitative variables were expressed as medians and quartiles. Logistic regression analysis was used to determine the association of the dependent variables and CAL ≥ 3 mm, respectively. Mean values of three independent observations from each individual were used in the calculations. Differences between the effect of the two different blends of mouth rinse on the levels of salivary biomarkers and *A. actinomycetemcomitans* were evaluated by Mann Whitney *U* test. Confidence interval and *P* values were based on the Wald statistics. A level of significance of 5% (*P* < 0.05) was used for the analyses.

## 3 RESULTS

Clinical registrations of the study population showed that four out of the 22 individuals had ≥1 site of AL ≥ 3 mm (Table 4).

**Table 4:** Demographic and clinical data from the study population

Case no.	Age	Gender	Rinse	JP2	No. of teeth AL ≥ 3mm
**#69**	13	Female	Placebo	Pos	0
**#112**	14	Female	Active	Pos	**1**
**#125**	14	Female	Active	Pos	**2**
**#138**	14	Male	Placebo	Pos	0
**#191**	14	Male	Active	Pos	0
**#196**	13	Male	Placebo	Pos	0
**#211**	13	Female	Active	Pos	0
**#250**	14	Male	Placebo	Pos	0
**#261**	12	Female	Active	Pos	0
**#263**	13	Female	Placebo	Pos	0
**#277**	13	Female	Placebo	Pos	0
**#282**	14	Female	Active	Pos	0
**#286**	14	Male	Placebo	Pos	0
**#319**	13	Female	Active	Pos	0
**#324**	13	Female	Active	Pos	0
**#355**	14	Male	Placebo	Pos	0
**#386**	14	Male	Active	Pos	0
**#394**	13	Female	Placebo	Pos	**3**
**#395**	14	Female	Placebo	Pos	**10**
**#398**	13	Male	Placebo	Pos	0
**#428**	13	Male	Active	Pos	0
**#449**	14	Female	Placebo	Pos	0

Bold indicates presence of AL = 3 mm.

**Figure 1:**
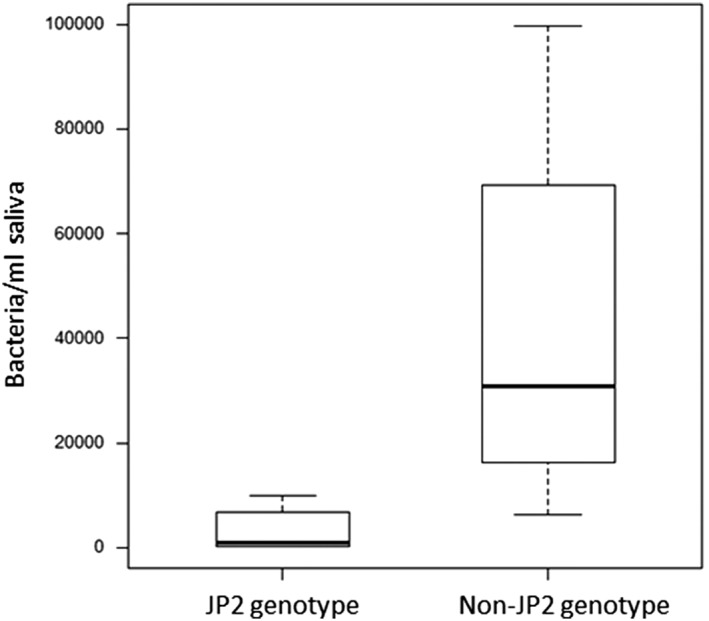
Levels of JP2 and non‐JP2 genotypes of *Aggregatibacter actinomycetemcomitans* in saliva from Moroccan adolescents. Medians and quartiles of three samples from 22 individuals are shown

**Figure 2:**
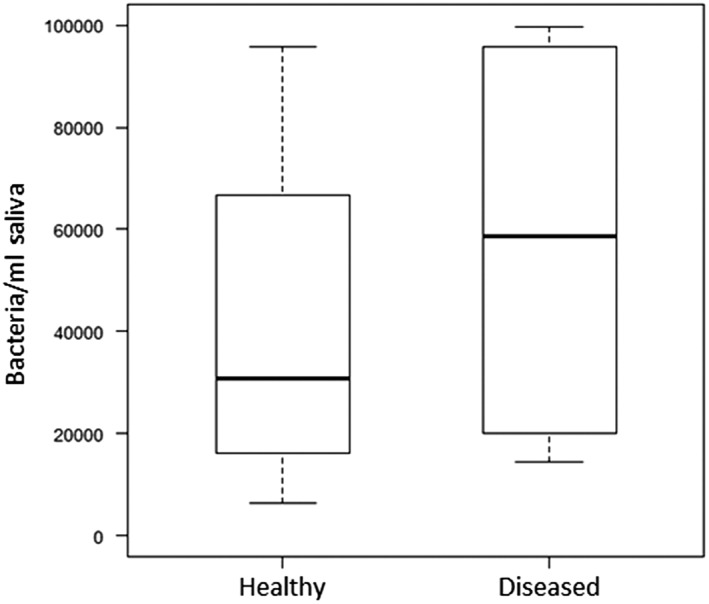
Levels of non‐JP2 genotype of *Aggregatibacter actinomycetemcomitans* in saliva from Moroccan adolescents, with or without clinical attachment loss (≥3 mm ≥ 1 tooth). Medians and quartiles of three samples from 18 healthy and four diseased individuals are shown

**Figure 3:**
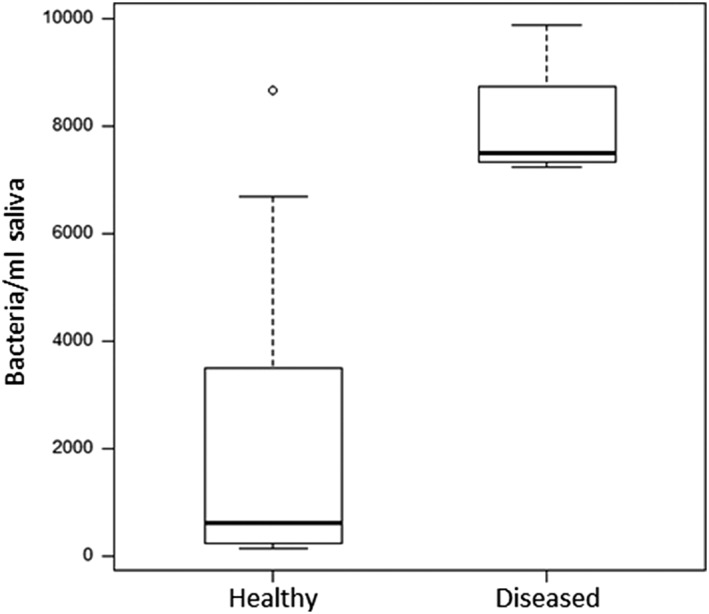
Levels of JP2 genotype of *Aggregatibacter actinomycetemcomitans* in saliva from Moroccan adolescents, with or without clinical attachment loss (≥3 mm ≥ 1 tooth). Medians and quartiles of three samples from 18 healthy and four diseased individuals are shown

Both the JP2 and the non‐JP2 genotype of *A. actinomycetemcomitans* could be detected in saliva of all individuals at least in one of the three samples collected during the study. The concentration of the non‐JP2 genotype was higher than for the JP2 genotype in the majority of the samples, and the average values in the saliva samples collected during the study are illustrated (Figure 1). The salivary levels of the non‐JP2 genotype were not significantly different between the periodontally healthy individuals and the diseased individuals (CAL ≥ 3 mm at ≥1 site; Figure 2). When the salivary levels of the JP2 genotype were compared in the two groups, the levels were significantly higher (*P* = 0.05) in the diseased group (Figure 3).

**Figure 4:**
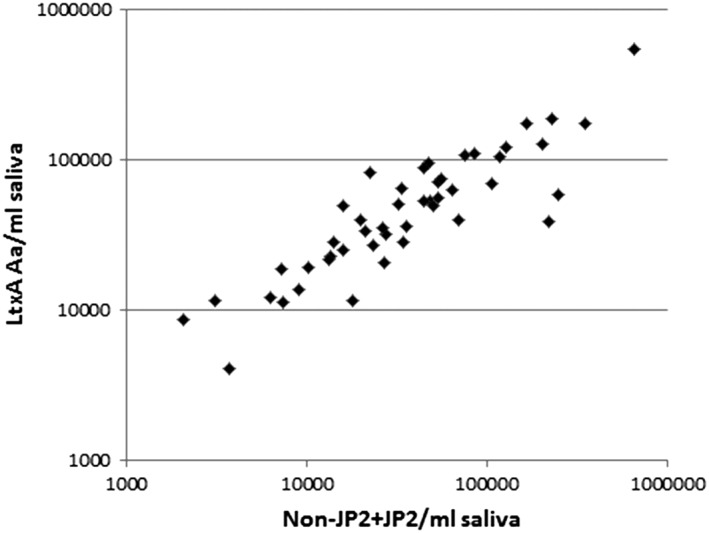
Levels of *Aggregatibacter actinomycetemcomitans* in saliva collected from Moroccan adolescents. Correlation of results from two different qPCR‐based methods is shown. JP2 and non JP2 genotype‐specific sequences within the leukotoxin promotor region (x‐axis) and sequences within *ltxA* (y‐axis) are targeted

The total levels of *A. actinomycetemcomitans* in the saliva samples were analyzed with two different setups, and similar results were achieved. The total levels of *A. actinomycetemcomitans* were achieved either by summarizing the PCR data of non‐JP2 and JP2 genotypes or by directly targeting a common gene (*ltxA*) for both genotypes in the PCR reaction. A comparison between the two methods, based on the results from all of the 66 samples, is illustrated and showed a significant correlation (*r* ^2^ = 0.808; Figure 4).

**Figure 5:**
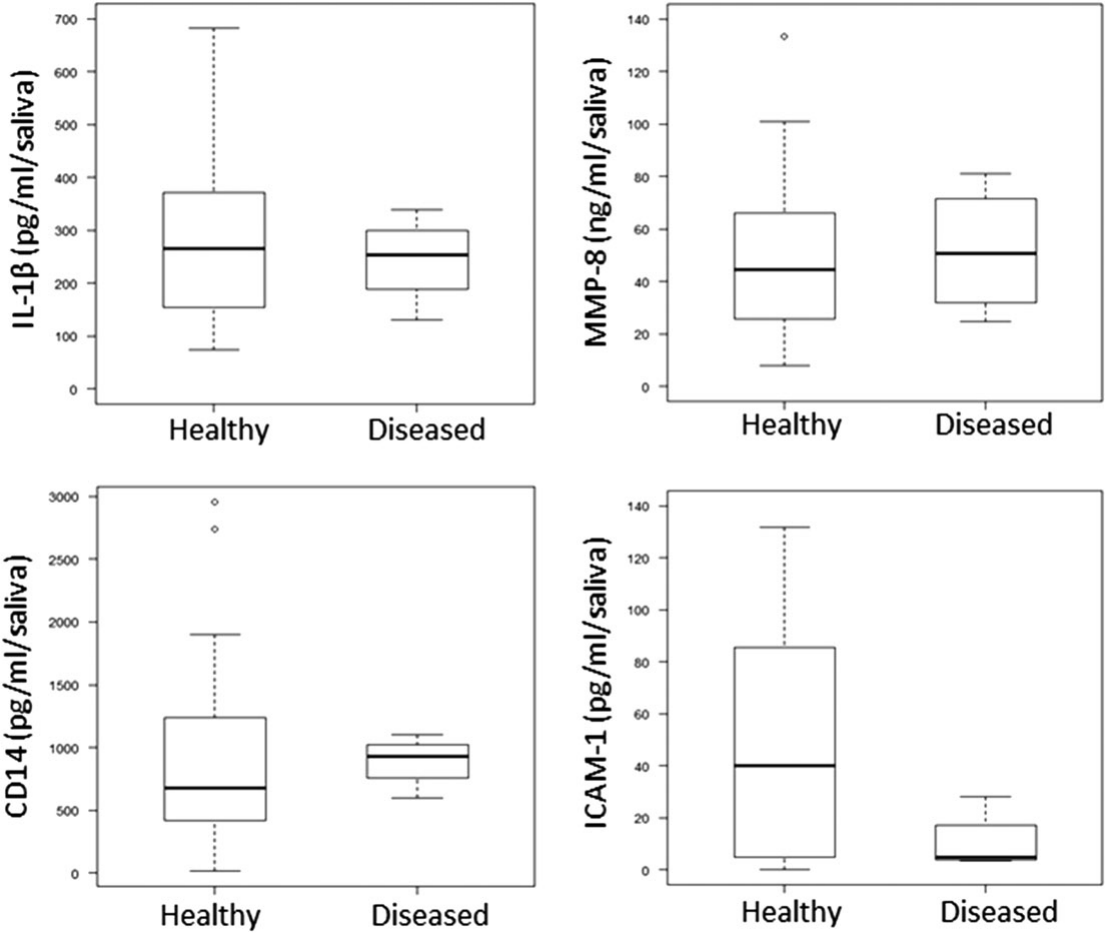
Concentration of inflammatory proteins, cytokines and intercellular adhesions molecules in saliva from Moroccan adolescents with or without clinical attachment loss (≥ 3 mm ≥ 1 tooth). Median and quartiles of three samples from healthy (n = 18) and diseased (n = 4) individuals are shown

The salivary concentrations of the inflammatory proteins IL‐1β, MMP‐8, sCD14, and sICAM‐1 were compared between the two groups of periodontally healthy or diseased individuals and showed that there were no significant differences between the two groups (Figure 5).

The use of active leukotoxin‐neutralizing mouth rinse during the 4‐week test period did not significantly change the salivary levels of *A. actinomycetemcomitans* or inflammatory proteins compared with the group that was administrated the placebo mouth rinse (Table 5). Despite these negative results, 69.6% of the students stated that gingival bleeding was improved after using the mouth rinse. This self‐reported improvement was independent of if the student used active or placebo mouth rinse. However, students' statements are subjective, and variables like plaque index and bleeding index should be evaluated in a future study on a larger population to help on having objective conclusions.

**Table 5:** Effect of active and placebo mouth rinse on salivary levels of the inflammatory proteins (IL‐1β and MMP‐8) and *A. actinomycetemcomitans* (JP2, non‐JP2, and total). Medians and [quartiles] from 10 individuals with active rinse and 12 individuals with placebo

	Rinse	IL‐1β (pg/ml)	MMP‐8 (ng/ml)	JP2 cells × 10^3^/ml	Non‐JP2 cells × 10^3^/ml	JP2 + nJP2 cells × 10^3^/ml
Baseline	Active	93.16 [26.96–323.76]	1.95 [0.37–5.48]	0.33 [0.098–1.030]	48.58 [16.88–127.47]	47.70 [11.71–77.99]
2 weeks		331.73 [191.44–547.03]	33.19 [2.1–91.23]	0.58 [0.16–28.389]	46.58 [24.30–81.02]	46.21 [27.00–117.86]
4 weeks		186.98 [153.16–517.24]	79.02 [45.42–120.22]	1.56 [0.464–12.74]	30.62 [18.81.117.57]	31.18 [18.87–148.16]
Baseline	Placebo	117.45 [47.58–291.72]	3.22 [0.48–8.81]	2.26 [0.61–8.75]	33.47 [6.25–56.4]	33.47 [13.88–69.44]
2 weeks		307.30 [193.45–467.77]	62.84 [23.52–88.06]	0.73 [0.27–84.04]	25.45 [9.92–82.78]	54.74 [11.53–109.80]
4 weeks		278.34 [142.42–388.92]	83.77 [50.44–121.89]	2.04 [0.25–61.92]	37.24 [12.62–115.67]	80.78 [156.55–222.77]

## 4 DISCUSSION

Salivary analyses on the young (12–15 years old) study population in the present study showed that *A. actinomycetemcomitans* of both the non‐JP2 and the JP2 genotypes could be detected in all individuals. The presence of the JP2‐genotype in saliva of all individuals was not surprising; based on that, the inclusion criteria for the studied adolescents was a previous detection of this genotype in samples from subgingival plaque. Despite this inclusion criterion, the salivary concentration of non‐JP2 genotype was much higher than that of the JP2 genotype in the majority of the samples. In four of the 22 individuals, CAL of ≥3 mm could be detected and these individuals were classified as periodontally diseased, in line with previous investigations (Haubek et al., 2008). When results from the periodontally diseased individuals were separated from that of the healthy, we found that the JP2 genotype was detected at high levels in saliva from the periodontally diseased individuals. This further strengthens the role of the JP2 genotype as a disease‐associated risk factor in this population, in line with previous studies (Haubek, 2010; Tsai et al., 2018). High levels of JP2 genotype *A. actinomycetemcomitans* in saliva may be a risk factor for disease onset but could also be a result of release of bacteria from diseased pockets. It has previously been shown that the presence of periodontal pathogens in subgingival plaque correlate with that found in saliva; however, these data were semiquantitative without specific quantification (Haririan et al., 2014; Kageyama et al., 2017).

Saliva has previously been used as a source for quantification of host inflammatory proteins associated to infection or inflammation (Bostanci & Bao, 2017). In the present study, we found that out of the periodontitis‐associated biomarkers that were quantified, no one of them were linked to disease. We have previously showed that the JP2 genotype is an efficient inducer of IL‐1β secretion from human macrophages (Kelk, Claesson, Chen, Sjöstedt, & Johansson, 2008). *A. actinomycetemcomitans* leukotoxin affects human macrophages by activating the inflammasome complex, which results in a massive release of bioactive IL‐1β (Johansson, 2011). It has previously been shown that periodontal pockets with high levels of *A. actinomycetemcomitans* are associated with enhanced IL‐1β concentrations in the gingival crevicular fluid (Kelk et al., 2008). However, high levels of JP2 genotypes of *A. actinomycetemcomitans* in saliva or occurrence of CAL did not significantly change the levels of the inflammatory proteins analyzed in saliva from individuals of the present study population. This indicates that AgP patients infected with the JP2 genotype of *A. actinomycetemcomitans* cannot be diagnosed by measuring levels of inflammatory proteins in saliva. We hypothesize that high level of the JP2 genotype in saliva may be a risk marker for development of CAL but is harmless until they infect the gingival margin. We show for the first time that high levels of the JP2 genotype in saliva is significantly associated with the presence of AL; however, further studies with longitudinal design are needed to confirm this hypothesis. Such longitudinal studies will also clarify if high salivary level of the JP2 genotype is a risk marker or a sign for the disease onset.

Treatment of aggressive forms of periodontitis is time‐consuming and expensive, both for the patient and the health care system (Teughels, Dhondt, Dekeyse, & Quirynen, 2014). An early diagnosis and onset of treatment are important factors for an optimal success rate for disease remission (American Academy of Pediatrics Committee on Child Abuse and Neglect 2008‐2009). Preventive strategies may be an alternative if high‐risk individuals can be identified before diseases onset. As a first step in aim to test preventive strategies, we performed a small clinical trial on JP2 positive adolescents that were given active or placebo leukotoxin‐neutralizing mouth rinse during a 4‐week period. The salivary levels of the JP2 and the non‐JP2 genotypes of *A. actinomycetemcomitans*, as well as the levels of inflammatory protein, were analyzed during the test period. The use of active mout rinse compared to the placebo mouth rinse did not show any significant differences in the significant effect on concentrations of *A. actinomycetemcomitans* or inflammatory proteins in the saliva. The lack of effect by leukotoxin neutralization may be that the active compounds in the mouth rinse did not reach the infectious site in the periodontal pocket. Concerning the healthy individuals, we suggest that *A. actinomycetemcomitans* in saliva or on oral mucosa is harmless without inducing an inflammatory response that can be neutralized. In order to determine if leukotoxin neutralization can protect against translocation of the bacterium from the mucosa to the gingival crevice, the study period needs to be extended to about 2 years. Previous longitudinal studies have shown that presence of highly leukotoxic *A. actinomycetemcomitans* in subgingival plaque of periodontally healthy adolescents significantly predict for CAL at a 2‐year follow‐up examination (Haubek et al., 2008; Höglund Åberg et al., 2014).

We can conclude from the results in the present study that saliva can be a suitable source for identification of carrier ship for both JP2 and non‐JP2 genotypes of *A. actinomycetemcomitans*. High levels of the JP2 genotype of *A. actinomycetemcomitans* were correlated to the occurrence of CAL; however, it should be noted that the study population was small. Finally, with the present test protocol with 4 weeks use of leukotoxin‐neutralizing mouth rinse, we could not see any significant effects on the salivary levels of *A. actinomycetemcomitans* or inflammatory proteins.

## CONFLICT OF INTEREST

The authors declare that there is no conflict of interest.
